# Mesenchymal Stem Cell Benefits Observed in Bone Marrow Failure and Acquired Aplastic Anemia

**DOI:** 10.1155/2017/8076529

**Published:** 2017-12-03

**Authors:** Vivian Fonseca Gonzaga, Cristiane Valverde Wenceslau, Gustavo Sabino Lisboa, Eduardo Osório Frare, Irina Kerkis

**Affiliations:** ^1^Laboratory of Genetics, Butantan Institute, São Paulo, SP, Brazil; ^2^Post-Graduate Program Interunity in Biotechnology, University of São Paulo, São Paulo, SP, Brazil; ^3^CellAvita Ltd, Valinhos, SP, Brazil

## Abstract

Acquired aplastic anemia (AA) is a type of bone marrow failure (BMF) syndrome characterized by partial or total bone marrow (BM) destruction resulting in peripheral blood (PB) pancytopenia, which is the reduction in the number of red blood cells (RBC) and white blood cells (WBC), as well as platelets (PLT). The first-line treatment option of AA is given by hematopoietic stem cell (HSCs) transplant and/or immunosuppressive (IS) drug administration. Some patients did not respond to the treatment and remain pancytopenic following IS drugs. The studies are in progress to test the efficacy of adoptive cellular therapies as mesenchymal stem cells (MSCs), which confer low immunogenicity and are reliable allogeneic transplants in refractory severe aplastic anemia (SAA) cases. Moreover, bone marrow stromal cells (BMSC) constitute an essential component of the hematopoietic niche, responsible for stimulating and enhancing the proliferation of HSCs by secreting regulatory molecules and cytokines, providing stimulus to natural BM microenvironment for hematopoiesis. This review summarizes scientific evidences of the hematopoiesis improvements after MSC transplant, observed in acquired AA/BMF animal models as well as in patients with acquired AA. Additionally, we discuss the direct and indirect contribution of MSCs to the pathogenesis of acquired AA.

## 1. Introduction

Red bone marrow (BM) is a gluey, complex, and heterogeneous tissue found in the medullary cavity of long bone and spongy bone cavities of the body. It is anatomically made up of the stromal cells (fibroblasts, adventitial reticular cells, adipocytes, and others) responsible for the tissue structure [1] and the parenchymal cells (hematopoietic cells—blood-producing cells) [2, 3]. To fabricate these blood-producing cells, BM contains a pool of hematopoietic stem cells (HSCs), which are self-renewing cells, differentiate into red (erythrocytes) and white (leukocytes) blood cells, and generate megakaryocytes and these produce platelets (PLT) [2–4]. Only mature hematopoietic cells enter the bloodstream. With age, red BM tends to be substituted with yellow BM, which is mostly made up of fat cells [5, 6].

BM stroma is a key element of hematopoiesis that provides the structural and physiological support for blood cell production. It also consists of a heterogeneous population of different cell types among which is a rare population of nonhematopoietic skeletal progenitor cells named bone marrow stromal cells (BMSC) [7, 8]. Red BM (hematopoietic marrow) and stroma are crucial components of the hematopoietic microenvironment as they interact and produce together—or individually—humoral growth and/or inhibitory factors necessary to maintain normal hematopoiesis, which is essential for life and human health.

BM can be susceptible to two types of failure syndromes: inherited or acquired. The inherited bone marrow failure (BMF) syndromes are a group of disorders usually diagnosed in childhood and passed down from parent to child through the association with some genetic abnormality [9], which may cause the aplastic anemia (AA) and cancer predisposition [10]. Young people and adults usually may develop the acquired BMF, which can be caused by different extrinsic and intrinsic factors including chemicals, irradiation, chemotherapy treatments, and immune system harms [11, 12].

Initially, BMF syndromes were denominated as “idiopathic AA” because at first, etiology was unknown. Nowadays, the term “AA” encompasses a heterogeneous BMF disorders which are characterized by BM cellular component ablation [13, 14].

Among BMF diseases, the acquired AA is more common. The treatment of acquired AA depends on the patient's age, health, and the severity of the disease. Treatment of moderate cases of acquired AA is indicated blood transfusions and supportive care with an antibiotic. However, many moderate cases may progress to severe AA (SAA) [10]. Therefore, to treat acquired SAA, HSC transplant from matched sibling donor is a matter of choice, which in some cases is satisfactorily effective [15]. It can be used in combination or not with immunosuppressive (IS) therapies. However, most patients have no access to immediate HSC transplant due to the lack of a matched sibling donor. Frequently, extensive time is needed to find a suitable unrelated donor for HSC transplant in SAA patients [16, 17].

Allogeneic transplant of MSCs can be a potential supplementary alternative to treat refractory SAA, since these cells are hypoimmunogenic, thus displaying low expression levels of human leukocyte antigen (HLA) class I, no expression of HLA class II [18]. Potentially, these cells may also be an addition to IS therapies because they possess broad immunomodulatory properties, secreting several biological molecules that influence both adaptive and innate immune responses [19]. Some studies showed that MSCs can prevent graft-versus-host disease (GVHD) and improve hematopoiesis when coinfused with HSCs [20, 21]. Hence, animal models have been developed to assess the response of MSCs in acquired AA as well as the hematologic cell amelioration [22–24] to find conditions to improve HSC transplant regimens or even to evaluate its own effect to reverse BMF and consequently to enhance survival rates of the patients.

This review aims to critically evaluate the potential of MSCs, focusing mainly of BMSC, on acquired BMF/AA in animal models and in recent AA reported clinical cases.

## 2. AA: Origin, Causes, Diagnostic, and Treatment

AA was first described in 1888 by Paul Erich merely as an “empty” BM with replacement by fat cells [25] and now is defined by decreased hematopoietic precursors in the BM, resulting in BM hypoplasia, peripheral blood (PB) pancytopenia, and precocious fat replacement [26, 27].

The etiology of BM precursor destruction remained elusive for decades [13]. Currently, heterogeneous origin of this disease is accepted. Some inherited disorders can damage the BM cells and lead to AA, mostly as Fanconi anemia (FA), Shwachman-Diamond syndrome (SDS), and dyskeratosis congenita (DC) [9]. The acquired AA can be induced by many different factors such as antineoplastic drugs, antibiotics, nonsteroidal anti-inflammatory drugs, and pesticides, as well as active viral infections (Epstein Barr, hepatitis virus, human immunodeficiency virus, and parvovirus) and radiation exposure [17, 28].

Most of the acquired AA is the result of an immuno-mediated process that leads to apoptosis of BM cells triggered by cytotoxic T cells [17, 29]. This process occurs as the result of an imbalance between CD8^+^ and CD4^+^ T cells, including T helper (Th) type 1 (Th1), Thtype 2 (Th2), regulatory T cells (Treg) and Th type 17 (Th17) cells, natural killer (NK) cells, and NK T cells. Besides that, there is abnormal production of cytokines including interferon- (IFN-) *γ*, tumor necrosis factor- (TNF-) *α*, and transforming growth factor (TGF) [30–34].

For acquired AA diagnostic, the pancytopenia is evaluated using three main criteria: neutrophil count lower than 0.5 × 10^9^ cells/L, platelet count lower than 20 × 10^9^ cells/L, and reticulocyte count lower than 1% [35]. Patients with acquired AA often present symptoms of anemia purpura or hemorrhage, and, frequently, infection that may worsen the symptoms [35].

The treatment of acquired AA depends on the severity of the disease. As already mentioned, treatment for moderate cases is based on red blood cell (RBC) transfusions to treat anemia, on platelet transfusions to prevent bleeding, and on supportive care in association with antibiotics [36] aiming to reestablish blood cell volume and prevent secondary infections.

Many moderate cases may progress to severe pancytopenia [35]. Moreover, for severe cases, the first-line treatment to date is HSC transplants from matched sibling donor, more efficient in young patients [15] and IS therapies, most commonly used due to lack of histocompatible sibling donors (HLA) and indicated for older patients [17].

Nevertheless, the success of HSC transplant is limited due to late complications, such as graft rejection and relapse due to resurgent autoimmune attack, and more often due to development of GVHD [15, 37], whereas lack of response, relapse, and clonal evolution limit the success of IS drugs [38].

## 3. MSCs and Mechanisms of Action

BMSCs are a natural component of stromal BM cellular environment, which are found at low frequency (0.001–0.01%) [39]. When isolated in vitro culture, they show fibroblast-like cell morphology with capacity to form colonies and are able to differentiate mainly into mesoderm derivatives. Moreover, only BMSCs have been shown to self-renew *in vivo* [40, 41].

More recently, similar mesenchymal stem cells (MSC) to BMSC were found in umbilical cord (UC) blood and Wharton's jelly [42], in adipose tissue (AT) [43] in dental pulp (DP) tissue [44], and in amniotic fluid [45] and other fetal and postnatal tissues [46, 47]. According to the International Society of Cellular Therapy (ISCT), MSCs, firstly, must be plastic-adherent when maintained in standard culture conditions. Second, they are characterized by expression of cell surface antigens (CD105, CD73, and CD90), lack of expression of CD34, CD45, CD14 or CD11b, CD79*α*, and HLA-DR surface molecules, and third, they showed the capacity to differentiate in vitro into adipocytes, osteoblasts, and chondroblasts [48]. However, MSCs derived from different sources have similar immune profile after in vitro culture expansion. On the other hand, it can possess a distinct differentiation potential and biological function, which depend on their embryonic and adult tissue origin [49, 50]. Moreover, profound differences in development potential between MSC sources were found, which are not dependent on donor age and may implicate with MSC clinical use [49, 50].

Paracrine mechanism of BMSC action was first evidenced by their capacity to support HSC growth and differentiation in vitro [51]. Furthermore, MSCs derived from adipose tissue (AT) have also been demonstrated as being able to support hematopoietic niche *in vitro* and *in vivo* [52]. Many studies focused on BMSC's ability to secrete a series of bioactive molecules, as cytokines and growth factors in response to injury into BM microenvironment [53–56]. BMSCs interact with HSC niche secreting such bioactive molecules to support proliferation and long-term growth of HSCs, thus influencing hematopoiesis [57]. Therefore, C-X-C motif chemokine ligand 12 (CXCL12) is responsible for regulation of adhesion, expansion, migration, and homing of HSCs. The Flt-3 ligand (FLT3LG), interleukin-6 (IL-6), and thrombopoietin (TPO) influence HSC proliferation, differentiation, and self-renewal, while stromal cell-derived factor 1 (SDF-1) reduces the production of inflammatory cytokines and chemokines [58–61].

In addition to paracrine effect, general MSCs demonstrate immunomodulatory activity *in vitro* [62, 63]. MSCs interact with various immune cells and secrete soluble mediators [53]. They express several adhesion molecules, including vascular cell adhesion molecule- (VCAM-) 1, intercellular cell adhesion molecule- (ICAM-) 1, and lymphocyte function-associated antigen- (LFA-) 3 involved in T cell interactions, which provide signaling of immunomodulatory response. MSCs suppress T cell proliferation and activation and regulate the differentiation of Th cells [64]. MSCs are capable to inhibit B cell activation, as well as, dendritic cells (DCs) and their precursor proliferation, differentiation, and maturation [65]. Moreover, MSCs modulate the immune responses by generation of Tregs to prevent immune intolerance. It is an important mechanism which could prevent GVHD [66]. Therefore, MSCs have significant clinical implications in BMF, such as acquired AA and related disorders [63].

## 4. BMSCs and AA

**Figure 1:**
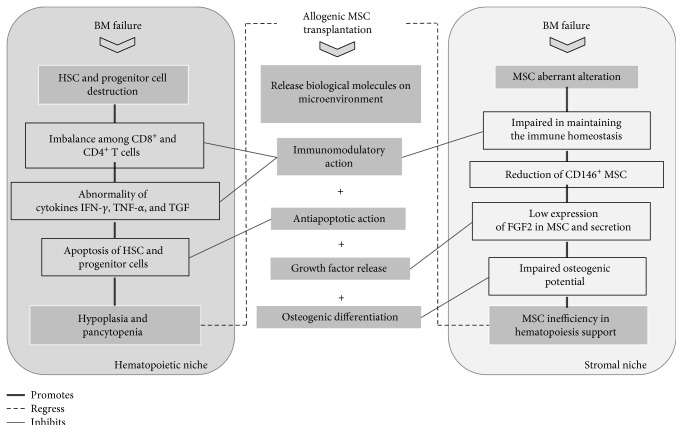
Effect of mesenchymal stem cell (MSC) transplant on bone marrow failure (BMF) etiology and progression. Acquired aplastic anemia (AA) is caused by destruction of hematopoietic stem cell (HSC) and progenitor cells associated with MSC abnormalities, caused by hematotoxic agents (drugs, chemical agents, radiation, and virus). These events lead to imbalance among CD8^+^ and CD4^+^ T cells and abnormal cytokine secretion, which correlates apoptosis of HSC and progenitor cells and consequently bone marrow (BM) aplasia and pancytopenia. Additionally, in turn of BM, imbalance aberrant alteration on MSC from BM niches arises. MSC aberrant alteration is observed by, impaired in maintaining the immune homeostasis, reduction of CD146^+^ MSC and low expression of FGF2 in MSC and its secretion, which lead to MSC inefficiency in hematopoiesis support and collaborate to progress of disease. According to the literature for AA, MSCs improve engraftment of HSC and prevent apoptosis in BM failures. BMF improvements occur as a result of MSC transplant through very similar mechanisms, such as immunomodulation, release growth factors, and osteogenic support. Although in vivo improvement on hematopoiesis was not demonstrated, several properties of MSCs, as well as its association with AA, justify the use of MSC in BM failures.

Although acquired AA is considered to affect mainly blood-producing cells, aplastic BM shows significant reduction in endosteal cells, vascular cells, and perivascular cells—pericytes [67, 68]. There are also growing evidences in the scientific literature that MSCs, which showed pericyte-like properties [69, 70]. When isolated from the SAA, patients are affected by this disease. They may present aberrant morphology, impaired osteogenic potential, changes in gene expression, and reduced ability to support hematopoiesis in vitro [71–73]. The number of CD146^+^ cells is reduced in aplastic BM [74]. This marker is expressed in bone marrow pericyte cells [70, 75–77] and BMSCs [76, 78–80] which can maintain the long-term repopulation potential of HSCs in vitro [81]. MSCs isolated from the patients with acquired AA patient were prone to differentiate into adipocytes rather than osteoblasts. These cells demonstrate downregulation of transcription factor (TF) GATA-2, which is expressed in hematopoietic progenitors, including early erythroid cells, mast cells, and megakaryocytes and overexpression of TF peroxisome proliferator-activated receptor gamma (PPAR*γ*) [82], which has multiple roles in MSCs obtained from AT of the patients with acquired AA [83, 84]. These alterations contribute to the abnormal AT deposit, thus affecting BM tissue remodeling and repair. A low expression level of basic fibroblastic growth factor 2 (FGF2) gene in BMSCs of AA patients was also reported [85, 86]. It is well known that BMSCs are the genuine source of FGF2, which directly influences the HSCs and their precursors in vitro [87, 88]. Furthermore, BMSCs from AA patients were impaired in maintaining the immune homeostasis associated with CD4^+^ T cells in vitro, which might cooperate with BMF [89]. In contrast to previous observations, one recent study shows that MSCs from patients diagnosed as moderate–severe AA did not present any alteration in morphology, osteogenic potential, gene expression, and ability to support hematopoiesis in vitro [90].  Figure 1 summarized recent studies that show the key role of MSCs in hematopoiesis and in AA pathogenesis, as well as demonstrate possible benefits from allogeneic MSC transplant in this disease.

## 5. Animal Models for the Study of Acquired AA

Animal models have greatly contributed to elucidate different aspects of BMF and acquired AA. Initially, the attempt to mimic AA has used exposure to toxic/chemical agents and pharmacological drugs that result in BMF through a direct toxic effect [13, 91–97], which were then replaced by physical and biological agents, as irradiation [22, 23] and lymph node infusion [98–102]. The administration of toxical/chemical and pharmacological agents results in BMF in attempt to mimic AA [13, 103]. However, the use of toxic drugs did not provide the immune-mediated destruction of the animal BM, which is commonly observed in human AA disease [13].

In turn, the model which employs infusion of lymph node cells in preirradiated animal shares many pathophysiological features with human immune-mediated AA. These animals develop BM hypoplasia rapidly, which is followed by severe peripheral pancytopenia, adipose cell invasion in BM, and hematopoietic cell reduction [102]. The changes in T lymphocyte subsets and IFN-*γ* ratio also occur [99].

Irradiation alone also causes BMF in animals. HSCs and committed BM progenitor cells present rapid cell turnover, thus being more sensitive to irradiation, when compared with other cell types [104–106]. The total body irradiation (TBI) using different doses of gamma irradiation ranging from two to eight grays (Gy) induces high expression of the apoptosis regulator gene (BAX gene) causing rapid lymphocyte death [107]. Low doses, around 2 Gy cobalt-60 gamma rays, result in decreased lymphocyte concentration and immune suppression in mouse. Medium and high doses of radiation (5 to 8 Gy) lead to BMF, neutropenia, thrombocytopenia, and anemia, as well as to low count of colony-forming unit granulocyte/macrophage (CFU-GM) and colony-forming unit fibroblast (CFU-f) [22]. Higher (above 8 Gy) doses of radiation may cause lethal hemorrhage or infections and death [108]. Therefore, care should be taken to choose the dose of radiation to induce BMF with minimized side effects and eventually low death incidence.

The murine models mimicking AA have improved over time. The development of an immune-mediated model in the destruction of the BM was important, being more closely modeling human AA [13]. However, human AA is extremely heterogeneous disease. Therefore, different stem cell therapeutic strategies, as source of the stem cell, doses, concentration, and periodicity, as well as the administration route, should be considered to treat different AA pathologies [109, 110]. MSCs, in turn, although showed similar basic characteristics, have different embryonic origin that may reflect on their medicinal paracrine properties [111, 112].

## 6. Benefits of MSC Transplant in BMF and AA Animal Models

Despite the immune-mediated animal model being considered the closest to mimic the pathophysiology of human AA, most studies choose the BMF animal model, which has failure induction mainly from irradiation strategy, to assess the mechanism of MSCs. The term “bone marrow failure” encompasses any primary failure condition at the HSCs, resulting to the decrease of one or more circulating blood cell lineage [113].  Table 1 summarizes the current knowledge regarding MSC transplantation into BMF and AA animal models. In the literature, the studies have used mouse irradiation doses ranging from 4 Gy to 8 Gy [22–24, 100, 114–116]. In immune-mediated AA animal model, the preirradiated (4 Gy) mouse received 1 × 10^6^ lymph node cells to induce acquired AA [98, 100]. MSCs from different sources were used in order to assess its possible therapeutic benefits, such as BMSC [22], umbilical cord-derived mesenchymal stem cells (UC-MSCs) [23, 24], adipose-derived mesenchymal stem cell (AD-MSCs) [115, 116], and multiplacenta-pooled cells, which contain MSCs derived from placenta, umbilical cord (UC), and UC blood [100]. Additionally, MSCs were infused in AA animal model in combination with HSCs [115] or extracellular superoxide dismutase (ECSOD), which is an extracellular searcher of superoxide (O_2_^·−^) and the main regulator of nitric oxide (NO) in the blood vessel wall and other organs [23]. On the majority of published works, a single MSC transplant was used and doses ranged from 1 × 10^6^ to 2.5 × 10^7^ cells per mouse. The cells were mostly administrated by endovenous (EV) [22, 23, 98, 116] and less by intraperitoneal (IP) route [100]. Only one study analyzed and showed engraftment of MSCs in BM after EV route [23]. Most in vivo studies did not use IS drugs before or during cell transplant [22, 24, 100, 114–116].

The preclinical studies report the increase of the levels of WBC [22, 24, 98], PTL [22, 23], and hemoglobin in PB [23, 100] after MSC transplant in comparison with the BMF control group that did not receive MSCs. Different studies show BM recovery and demonstrate an increased number of BM cells in vivo [22–24, 116] as well as an increased CFU-f [22, 116] and CFU-GM in vitro capacity [22, 98, 114]. Additionally, an increased megakaryocyte concentration was observed in BM after MSC transplantation [23, 116]. Besides, the increase of PB and BM cells, hematopoietic cytokines, as FLT3LG and TGF-beta1, was reported. These cytokines, which are secreted by MSCs, are important to HSC proliferation and differentiation process [24].

Although TBI led to HSCs and progenitor cells in BM apoptosis, few reports showed MSCs' antiapoptotic effect on HSC and hematopoietic progenitor cell preservation [22, 116]. The exact mechanism of MSCs' antiapoptotic effect is still under investigation. However, it has been shown that MSC transplant leads to a reduction of BAX gene expression in BM cells [116].

It is known that the increased levels of IFN-*γ* and TNF-*α* in irradiated mouse activate the Th1 and Th2 cells [24]. On the other hand, MSCs present immunoregulatory properties that could be used to attenuate the imbalance of immunologic system after radiation exposure [114, 117]. Hence, one study showed that MSCs reduce irradiation-induced hematopoietic toxicity. MSCs improved lymphocyte-mediated inhibition of CFU-GM and induced additional immunoprotective effects by expanding the Tregs, regulating chemokine receptor expression, and promoting the Th1/Th2 balance toward anti-inflammatory Th2 polarization [114].

Recent publications focused on preclinical studies demonstrated that MSCs could recover BMF by its antiapoptotic and immunoregulatory properties [22, 23, 114, 116]. However, all these studies evaluated MSC benefits after short-term experiments (from 24 hours to 30 days post-MSC injection); therefore, long-term benefits and stability on MSC transplant still need to be assessed.

## 7. Transplant of MSC in AA Patients

The clinical use of the MSCs in the hematological diseases has received special attention because of their inhibitory effects on the proliferation and cytotoxic activity of immune system cells in patients, which developed GVHD in response to allogeneic HSC transplantation [118, 119]. In AA patient transplant of allogeneic BM—or UC—MSCs were performed alone or in combination with HSCs [120–125] (Table 2). Some studies are registered at the National Institutes of Health (NIH) clinical trial database [126, 127]. The patients enrolled in these clinical trials presented severe stage of AA disease and did not respond to IS therapy (exhibit refractory stages). In addition, many patients prior to MSC transplant had already received treatment with HSC or BM cell transplant without clinical amelioration [121–123, 125]. In these studies, MSC doses ranged from 1 × 10^6^/kg to 1 × 10^7^/kg per transplant and the patients received one to two transplants per month.

Most of the studies did not evaluate whether MSCs engraft into host BM after EV transplant [121, 123–125]. Only one study showed MSC chimerism in a patient's BM microenvironment after MSC transplant. The chimerism study was performed by real-time PCR for the SRY gene for detection of male DNA in whole BM sample from a woman patient. This study showed improvement of BM stromal niche in a patient with SAA refractory to ATG and cyclosporine who was ineligible for allogeneic HSCT. After receiving two allogeneic transplants of MSCs, the biopsy demonstrated reduction of necrotic areas, but the BM improvement was not observed [120].

Cotransplant of MSC and HSC therapy also shows hematopoietic recovery in AA in humans [123, 128]. Six patients were treated, and two of them presented a hematopoietic recovery in both BM and PB three months after transplant [123].

All clinical studies used immunosuppression protocol [120–125]. In spite of this, some patients manifested adverse events such as mild self-limited febrile reactions, headaches, hypoxemia, mild dyspnea, and diarrhea after MSC transplant. All these adverse events were observed during or after MSC infusions and were mild and self-limited [123, 125]. Three studies reported a few deaths of SAA patients after the second or third MSC transplant alone [120, 125] or combined with HSCs [124]. However, these deaths occurred as a result of natural complications of AA disease [120, 124, 125]. Besides, no study reported occurrence of tumor after MSC transplant during the follow-up studies.

There are few clinical cases which use the therapy with MSCs on the AA disease, and then only in the most severe cases which did not respond to conventional treatment. And these studies show that the treatment was safe, but not enough to alone recover the BM. This observation can suggest that therapy with MSCs is promising but still needs to be in combination with HSC transplant [120, 125].

## 8. Final Considerations

Regulatory agencies require that investigators provide robust data on in vivo efficiency of new biological products. They recommend the use of well-characterized animal models to predict the response in humans. In general, transgenic animals are more indicated for this purpose. However, as we mentioned above, in the case of acquired AA, an immune-mediated animal model is well accepted [102]. MSC therapeutic potential was assessed using two BMF models: immune-mediated and irradiation-induced model [22–24, 100, 114–116]. Although these studies helped to demonstrate the several benefits of MSCs on acquired AA, they present limitation—natural reversibility of AA pathogenesis following long periods of evaluation [129].

On the other hand, clinical studies which use MSCs, demonstrate that patients with a very severe form of AA were enrolled, as well as each study includes very limited number of patients. Another drawback of clinical studies includes the use of IS drugs [120–125] which hinder the interpretation of results, as these drugs may ameliorate and even recover BMF alone [130, 131]. In addition, IS drugs could negatively influence the therapeutic action of the MSCs [132].

## Figures and Tables

**Table 1:** MSC transplantation in BMF and AA animal models.

Reference	Mice model	Gender	Age (weeks)	BMF induction method	Cell source	MSC profile	Number of transplanted MSC	A.R.	Evidence of MSC efficacy
[22]	Balb/c	F	6-7	Irradiation (5.5 Gy)	BM-MSC	CD34^−^, CD45^−^, CD105^+^, CD29^+^, CD44^+^, and Sca-1^+^	2.5 × 10^7^	EV	↑ WBC and PLT in PB, CFU-F, and CFU-GM ↑ BMC ↓ apoptotic cells in BM
[23]	Balb/c	M	6	Irradiation (5.8 Gy)	UC-MSC + ECSOD	CD14^−^, CD73^+^, CD90^+^, CD105^+^, CD44^+^, CD29^+^, CD34^−^, CD45^−^, CD19^−^, and HLA-DR^−^	1 × 10^6^	EV	↑ WBC, PLT, RBC, HB Attenuate upregulation of apoptotic genes (p16, p21, p53, and NOX4) ↑ BM cells and megakaryocyte ↓ apoptotic cells
[24]	Balb/c	F	6	Irradiation (7 Gy)	hUC-MSC	CD105^+^ and CD34^−^	NI	NI	↑ WBC in PB and BMC ↑ levels of hematopoietic cytokines (Flt3L and TGF-*β*1)
[100]	Balb/c	F	8	Irradiation (4 Gy) + lymph node cell infusion	Multiplacentas pooled cells	NI	1 × 10^7^	IP	Higher survival ↑ HB
[114]	Balb/c	NI	NI	Irradiation (8 Gy)	CB-MSC	CD45^−^, CD34^+^, CD29^+^, CD44^+^, CD117^−^, and Sca-1^+^	NI	NI	↑ survival and gain body weight ↑CFU-GM ↑ Treg cells ↓ suppressed Th1 immunity and regulation of T cell chemokine receptor expressions (CCR7 upregulation and CXCR3/CCR5 downregulation)
[115]	B6D2F1	NI	10–12	Irradiation (5–7 Gy)	HSC coinfusion of AD-MSC	CD29^+^, CD44^+^, CD73^+^, CD90.2^+^, CD105^+^, CD106^+^, CD144^+^, CD166^+^, CD34^−^, CD45.1^−^, CD80^−^, and Sca-1^−^	1 × 10^6^	NI	BM reconstitution Facilitating and homing of HSC to recipient BM
[116]	Balb/c	M	6–8	Irradiation (4 Gy)	AD-MSC	CD29^+^, CD31^−^, CD34^−^, CD45^−^, and CD90^+^	1 × 10^6^	EV	↑ CFU-F, CFU-MK, and megakaryocytes in BM (cd41^+^ cells) Recovery of BM cells ↓ apoptotic cells

A.R.: administration route; NI: noninformed; F: female; M: male; BMF: bone marrow failure; MSC: mesenchymal stem cell; BMSC: bone marrow stromal cell; hUC-MSC: human umbilical cord-derived mesenchymal stem cell; UC-MSC: umbilical cord-derived mesenchymal stem cell; CB-MSC: compact bone-derived mesenchymal stem cell; ECSOD: extracellular superoxide dismutase; HSC: hematopoietic stem cell; AD-MSC: adipose-derived mesenchymal stem cell; CD: cluster differentiation; Sca-1: stem cell antigen-1; HLA-DR: human leucocyte antigen-D related; EV: endovenous infusion; IP: intraperitoneal infusion; WBC: white blood cell; PLT: platelet; PB: peripheral blood; CFU-F: colony-forming unit fibroblast; CFU-GM: colony-forming unit granulocyte-macrophage; BMC: bone marrow cell; BM: bone marrow; Flt3L: FMS-like tyrosine kinase 3 ligand; TGF-*β*: transforming growth factor beta; CCR7: C-C chemokine receptor type 7; CXCR3: C-X-C motif receptor 3; CCR5: C-C chemokine receptor type 5; RBC: red blood cell; HB: hemoglobin; NOX 4: nicotinamide adenine dinucleotide phosphate oxidase 4; CFU-MK: colony-forming unit megakaryocytes.

**Table 2:** MSC transplantation in reported clinical cases with SAA.

Reference	Cell source	MSC profile	Number of patients	Age of patient (years)	Gender	Disease stage	Previous HSC or BM transplant	Number of transplants	A.R.	Number of MSC/kg	Immunossupressive treatment	Adverse event	Follow-up (months)	Death (patients)	Evidence of MSC efficacy
[120]	BMSC	SH2^+^, SH3^+^, CD34^−^, and CD45^−^	1	68	F	Refractory SAA	−	2	EV	2 × 10^6^ and 6 × 10^6^	CsA	NI	NI	1	MSC engraftment Partial recovery of BM stromal niche ↑ CFU-F (*n* = 1)
[121]	BMSC	NI	1	26	M	SAA	+	2	EV	1 × 10^6^	CTX, ATG, TBI, FAMP, and ALS	NI	NI	None	Partial recovery of BM niche (*n* = 1)
[122]	UC-MSC coinjection HSC	CD13^+^, CD29^+^, CD44^+^, CD73^+^, CD90^+^, CD105^+^, CD14^−^, and CD31^−^, CD34^−^, CD45^−^, and HLA-DR^−^	2	11 and 13	F	SAA	+	1	NI	1 × 10^6^	CsA, ATG, and Methylprednisolone	None	NI	None	Enhance the HSC engraftment (*n* = 2)
[123]	BMSC	CD29^+^, CD73^+^, CD90^+^ CD105^+^, CD34^−^, CD45^−^, and CD14^−^	14/4	16–56	M/F	Refractory SAA or NSAA	−	4–6	EV	6 × 10^5^	CsA and ATG	Transient fever and headache (*n* = 2)	12	None	Recovery of three hematopoietic cell line (*n* = 2) Recovery RBC and hemoglobin level (*n* = 2) Recovery PLT (*n* = 2)
[124]	UC-MSC coinjection HSC	VEGFR2/Flk1^+^, CD166^+^ CD105^+^, CD44^+^, CD29^+^, and HLA class I^+^ CD34^−^, CD45^−^, CD14^−^, and HLA class II^−^	14/23	15	F/M	SAA	+	1	EV	1 × 10^6^	CTX, FAMP, and ATG	None	60	9	↑Neutrophil ↑PLT Enhance the HSC homing and engrafting
[125]	BMSC	CD73^+^, CD90^+^, CD 29^+^, CD13^+^, CD44^+^, CD49e^+^, STRO1^+^, HLA class I^+^;CD34^−^, CD14^−^, CD45^−^, glycophorin A^−^, CD31^−^, cadherin^−^, KDR^−^, and HLA class II^−^	9	19–50	F and M	Refractory SAA or NSAA	−	5	EV	2.7 × 10^6^	CsA and ATG	Fever, hypoxemia, mild dyspnea, and diarrhea	20	4	Partial hematologic response (*n* = 2)

MSC: mesenchymal stem cells; BMSC: bone marrow stromal cell; hUC-MSC: human umbilical cord-derived mesenchymal stem cell; HSC: hematopoietic stem cells; CD: cluster differentiation; HLA-DR: human leucocyte antigen-D related; HLA: human leucocyte antigen; VEGFR2: vascular endothelial growth factor receptor 2; SAA: severe aplastic anemia; NSAA: nonsevere aplastic anemia; EV: endovenous infusion; CsA: cyclosporine A; ATG: antithymocyte globulin; ASL: antilymphocyte serum; TBI: total body irradiation; FAMP: fludarabine; BM: bone marrow; CFU-F: colony-forming unit fibroblast; RBC: red blood cell; PLT: platelets; NI: noninformed; CTX: cyclophosphamide; SH2: Src homology 2; SH3: Src homology 3; VEGFR2/Flk1: vascular endothelial growth factor receptor 2.

## References

[1] Payne M. W. C., Uhthoff H. K., Trudel G. (2007). Anemia of immobility: caused by adipocyte accumulation in bone marrow. *Medical Hypotheses*.

[2] Muller-Sieburg C. E., Whitlock C. A., Weissman I. L. (1986). Isolation of two early B lymphocyte progenitors from mouse marrow: a committed pre-pre-B cell and a clonogenic Thy-1^lo^ hematopoietic stem cell. *Cell*.

[3] Birbrair A., Frenette P. S. (2016). Niche heterogeneity in the bone marrow. *Hematopoietic Stem Cells Ix*.

[4] Zon L. I. (2008). Intrinsic and extrinsic control of haematopoietic stem-cell self-renewal. *Nature*.

[5] Taccone A., Oddone M., Dellacqua A., Occhi M., Ciccone M. A. (1995). MRI road-map of normal age-related bone-marrow. II. Thorax, pelvis and extremities. *Pediatric Radiology*.

[6] Moore S. G., Dawson K. L. (1990). Red and yellow marrow in the femur: age-related changes in appearance at MR imaging. *Radiology*.

[7] Owen M., Friedenstein A. J. (1988). Stromal stem-cells: marrow-derived osteogenic precursors. *Ciba Foundation Symposia*.

[8] Caplan A. I. (1991). Mesenchymal stem-cells. *Journal of Orthopaedic Research*.

[9] Dokal I., Vulliamy T. (2008). Inherited aplastic anaemias/bone marrow failure syndromes. *Blood Reviews*.

[10] Shimamura A., Alter B. P. (2010). Pathophysiology and management of inherited bone marrow failure syndromes. *Blood Reviews*.

[11] Jacobs P. (1995). Bone marrow failure: pathophysiology and management. *Disease-a-Month*.

[12] Young N. S., Young N. S., Alter B. P. (1994). Drugs and chemicals. *Aplastic Anemia: Acquired and Inherited*.

[13] Scheinberg P., Chen J. C. (2013). Aplastic anemia: what have we learned from animal models and from the clinic. *Seminars in Hematology*.

[14] Ahmed M., Dokal I. (2009). Understanding aplastic anaemia/bone-marrow failure syndromes. *Paediatrics and Child Health*.

[15] DeZern A. E., Brodsky R. A. (2011). Clinical management of aplastic anemia. *Expert Review of Hematology*.

[16] Margolis D. A., Casper J. T. (2000). Alternative-donor hematopoietic stem-cell transplantation for severe aplastic anemia. *Seminars in Hematology*.

[17] Young N. S., Calado R. T., Scheinberg P. (2006). Current concepts in the pathophysiology and treatment of aplastic anemia. *Blood*.

[18] Pang Y., Xiao H. W., Zhang H. (2017). Allogeneic bone marrow-derived mesenchymal stromal cells expanded in vitro for treatment of aplastic anemia: a multicenter phase II trial. *Stem Cells Translational Medicine*.

[19] Le Blanc K., Tammik L., Sundberg B., Haynesworth S. E., Ringden O. (2003). Mesenchymal stem cells inhibit and stimulate mixed lymphocyte cultures and mitogenic responses independently of the major histocompatibility complex. *Scandinavian Journal of Immunology*.

[20] Kuzmina L. A., Petinati N. A., Parovichnikova E. N. (2012). Multipotent mesenchymal stromal cells for the prophylaxis of acute graft-versus-host disease—a phase II study. *Stem Cells International*.

[21] Zhao K., Liu Q. F. (2016). The clinical application of mesenchymal stromal cells in hematopoietic stem cell transplantation. *Journal of Hematology & Oncology*.

[22] Hu K. X., Sun Q. Y., Guo M., Ai H. S. (2010). The radiation protection and therapy effects of mesenchymal stem cells in mice with acute radiation injury. *British Journal of Radiology*.

[23] Gan J. Y., Meng F. W., Zhou X. (2015). Hematopoietic recovery of acute radiation syndrome by human superoxide dismutase-expressing umbilical cord mesenchymal stromal cells. *Cytotherapy*.

[24] Shim S., Lee S. B., Lee J. G. (2013). Mitigating effects of hUCB-MSCs on the hematopoietic syndrome resulting from total body irradiation. *Experimental Hematology*.

[25] Ehrlich P. (1888). Ueber einen fall von anämie mit bemerkungen über regenerative veränderungen des knochenmarks. *Charité-Annalen*.

[26] Gewirtz A. M., Hoffman R. (1985). Current considerations of the etiology of aplastic-anemia. *Crc Critical Reviews in Oncology/Hematology*.

[27] Wintrobe M. M., Richard Lee G., Bogs D. R. (1974). Pancytopenia, aplastic anemia and pure red cell aplasia. *Clinical Hematology*.

[28] Gaman A., Gaman G., Bold A. (2009). Acquired aplastic anemia: correlation between etiology, pathophysiology, bone marrow histology and prognosis factors. *Romanian Journal of Morphology and Embryology*.

[29] Young N. S., Maciejewski J. (1997). Mechanisms of disease - the pathophysiology of acquired aplastic anemia. *New England Journal of Medicine*.

[30] Zoumbos N. C., Gascon P., Djeu J. Y., Young N. S. (1985). Interferon is a mediator of hematopoietic suppression in aplastic-anemia in vitro and possibly in vivo. *Proceedings of the National Academy of Sciences of the United States of America*.

[31] Sloand E., Kim S., Maciejewski J. P., Tisdale J., Follmann D., Young N. S. (2002). Intracellular interferon-γ in circulating and marrow T cells detected by flow cytometry and the response to immunosuppressive therapy in patients with aplastic anemia. *Blood*.

[32] Dubey S., Shukla P., Nityanand S. (2005). Expression of interferon-γ and tumor necrosis factor-α in bone marrow T cells and their levels in bone marrow plasma in patients with aplastic anemia. *Annals of Hematology*.

[33] Zeng W. H., Miyazato A., Chen G. B., Kajigaya S., Young N. S., Maciejewski J. P. (2006). Interferon-γ-induced gene expression in CD34 cells: identification of pathologic cytokine-specific signature profiles. *Blood*.

[34] Li J. P., Zheng C. L., Han Z. C. (2010). Abnormal immunity and stem/progenitor cells in acquired aplastic anemia. *Critical Reviews in Oncology Hematology*.

[35] Young N. S. (2002). Acquired aplastic anemia. *Annals of Internal Medicine*.

[36] Scheinberg P., Young N. S. (2012). How I treat acquired aplastic anemia. *Blood*.

[37] Miano M., Dufour C. (2015). The diagnosis and treatment of aplastic anemia: a review. *International Journal of Hematology*.

[38] Scheinberg P. (2012). Aplastic anemia: therapeutic updates in immunosuppression and transplantation. *Hematology-American Society Hematology Education Program*.

[39] Friedenstein A. J., Petrakova K. V., Kurolesova A. I., Frolova G. P. (1968). Heterotopic transplants of bone marrow. Analysis of precursor cells for osteogenic and hematopoietic tissues. *Transplantation*.

[40] Caplan A. I., Dennis J. E. (1996). Mesenchymal stem cell progenitor cascade. *Matrix Biology*.

[41] Caplan A. I. (2007). Adult mesenchymal stem cells for tissue engineering versus regenerative medicine. *Journal of Cellular Physiology*.

[42] Wang H. S., Hung S. C., Peng S. T. (2004). Mesenchymal stem cells in the Wharton’s jelly of the human umbilical cord. *Stem Cells*.

[43] Zuk P. A., Zhu M., Mizuno H. (2001). Multilineage cells from human adipose tissue: implications for cell-based therapies. *Tissue Engineering*.

[44] Kerkis I., Kerkis A., Dozortsev D. (2006). Isolation and characterization of a population of immature dental pulp stem cells expressing OCT-4 and other embryonic stem cell markers. *Cells, Tissues, Organs*.

[45] In 't Anker P. S., Scherjon S. A., Kleijburg-van der Keur C. (2003). Amniotic fluid as a novel source of mesenchymal stem cells for therapeutic transplantation. *Blood*.

[46] Wenceslau C. V., Miglino M. A., Martins D. S. (2011). Mesenchymal progenitor cells from canine fetal tissues: yolk sac, liver, and bone marrow. *Tissue Engineering Part A*.

[47] Shin K. S., Na K. H., Lee H. J. (2009). Characterization of fetal tissue-derived mesenchymal stem cells. *International Journal of Stem Cells*.

[48] Dominici M., Le Blanc K., Mueller I. (2006). Minimal criteria for defining multipotent mesenchymal stromal cells. The International Society for Cellular Therapy position statement. *Cytotherapy*.

[49] Sacchetti B., Funari A., Remoli C. (2016). No identical “mesenchymal stem cells” at different times and sites: human committed progenitors of distinct origin and differentiation potential are incorporated as adventitial cells in microvessels. *Stem Cell Reports*.

[50] Reinisch A., Etchart N., Thomas D. (2015). Epigenetic and in vivo comparison of diverse MSC sources reveals an endochondral signature for human hematopoietic niche formation. *Blood*.

[51] Dexter T. M. (1982). Stromal cell associated haemopoiesis. *Journal of Cell Physiology Supplement*.

[52] Nakao N., Nakayama T., Yahata T. (2010). Adipose tissue-derived mesenchymal stem cells facilitate hematopoiesis *in vitro* and *in vivo* advantages over bone marrow-derived mesenchymal stem cells. *American Journal of Pathology*.

[53] Caplan A. I., Dennis J. E. (2006). Mesenchymal stem cells as trophic mediators. *Journal of Cellular Biochemistry*.

[54] Hofer H. R., Tuan R. S. (2016). Secreted trophic factors of mesenchymal stem cells support neurovascular and musculoskeletal therapies. *Stem Cell Research & Therapy*.

[55] Baraniak P. R., McDevitt T. C. (2010). Stem cell paracrine actions and tissue regeneration. *Regenerative Medicine*.

[56] Rennert R. C., Sorkin M., Garg R. K., Gurtner G. C. (2012). Stem cell recruitment after injury: lessons for regenerative medicine. *Regenerative Medicine*.

[57] Majumdar M. K., Thiede M. A., Haynesworth S. E., Bruder S. P., Gerson S. L. (2000). Human marrow-derived mesenchymal stem cells (MSCs) express hematopoietic cytokines and support long-term hematopoiesis when differentiated toward stromal and osteogenic lineages. *Journal of Hematotherapy & Stem Cell Research*.

[58] Meirelles L. D., Fontes A. M., Covas D. T., Caplan A. I. (2009). Mechanisms involved in the therapeutic properties of mesenchymal stem cells. *Cytokine & Growth Factor Reviews*.

[59] Sugiyama T., Kohara H., Noda M., Nagasawa T. (2006). Maintenance of the hematopoietic stem cell pool by CXCL12-CXCR4 chemokine signaling in bone marrow stromal cell niches. *Immunity*.

[60] Zhang J. W., Li L. H. (2008). Stem cell niche: microenvironment and beyond. *Journal of Biological Chemistry*.

[61] da Silva C. L., Goncalves R., Crapnell K. B., Cabral J. M. S., Zanjani E. D., Almeida-Porada G. (2005). A human stromal-based serum-free culture system supports the ex vivo expansion/maintenance of bone marrow and cord blood hematopoietic stem/progenitor cells. *Experimental Hematology*.

[62] Gao F., Chiu S. M., Motan D. A. L. (2016). Mesenchymal stem cells and immunomodulation: current status and future prospects. *Cell Death & Disease*.

[63] Zhao Q., Ren H., Han Z. (2016). Mesenchymal stem cells: immunomodulatory capability and clinical potential in immune diseases. *Journal of Cellular Immunotherapy*.

[64] Bartholomew A., Sturgeon C., Siatskas M. (2002). Mesenchymal stem cells suppress lymphocyte proliferation in vitro and prolong skin graft survival in vivo. *Experimental Hematology*.

[65] Franquesa M., Hoogduijn M. J., Bestard O., Grinyo J. M. (2012). Immunomodulatory effect of mesenchymal stem cells on B cells. *Frontiers in Immunology*.

[66] Selmani Z., Naji A., Zidi I. (2008). Human leukocyte antigen-G5 secretion by human mesenchymal stem cells is required to suppress T lymphocyte and natural killer function and to induce CD4^+^CD25^high^FOXP3^+^ regulatory T cells. *Stem Cells*.

[67] Juneja H. S., Gardner F. H. (1985). Functionally abnormal marrow stromal cells in aplastic-anemia. *Experimental Hematology*.

[68] Park M., Park C. J., Jang S. (2015). Reduced expression of osteonectin and increased natural killer cells may contribute to the pathophysiology of aplastic anemia. *Applied Immunohistochemistry & Molecular Morphology*.

[69] Caplan A. I. (2008). All MSCs are pericytes?. *Cell Stem Cell*.

[70] Crisan M., Yap S., Casteilla L. (2008). A perivascular origin for mesenchymal stem cells in multiple human organs. *Cell Stem Cell*.

[71] Li W. X., Fu J. X., Wang Y., Shi W. B., Zhang X. G. (2005). Expression of membrane-bound IL-15 by bone marrow fibroblast-like stromal cells in aplastic anemia. *International Immunology*.

[72] Li J. P., Yang S. G., Lu S. H. (2012). Differential gene expression profile associated with the abnormality of bone marrow mesenchymal stem cells in aplastic anemia. *PLoS One*.

[73] Chao Y. H., Peng C. T., Harn H. J., Chan C. K., Wu K. H. (2010). Poor potential of proliferation and differentiation in bone marrow mesenchymal stem cells derived from children with severe aplastic anemia. *Annals of Hematology*.

[74] Wu L. L., Mo W. J., Zhang Y. P. (2015). Impairment of hematopoietic stem cell niches in patients with aplastic anemia. *International Journal of Hematology*.

[75] Crisan M., Corselli M., Chen W. C. W., Peault B. (2012). Perivascular cells for regenerative medicine. *Journal of Cellular and Molecular Medicine*.

[76] Covas D. T., Panepucci R. A., Fontes A. M. (2008). Multipotent mesenchymal stromal cells obtained from diverse human tissues share functional properties and gene-expression profile with CD146^+^ perivascular cells and fibroblasts. *Experimental Hematology*.

[77] Mangialardi G., Cordaro A., Madeddu P. (2016). The bone marrow pericyte: an orchestrator of vascular niche. *Regenerative Medicine*.

[78] Espagnolle N., Guilloton F., Deschaseaux F., Gadelorge M., Sensebe L., Bourin P. (2014). CD146 expression on mesenchymal stem cells is associated with their vascular smooth muscle commitment. *Journal of Cellular and Molecular Medicine*.

[79] Delorme B., Ringe J., Gallay N. (2008). Specific plasma membrane protein phenotype of culture-amplified and native human bone marrow mesenchymal stem cells. *Blood*.

[80] Shi S., Gronthos S. (2003). Perivascular niche of postnatal mesenchymal stem cells in human bone marrow and dental pulp. *Journal of Bone and Mineral Research*.

[81] Corselli M., Chin C. J., Parekh C. (2013). Perivascular support of human hematopoietic stem/progenitor cells. *Blood*.

[82] Ahmadian M., Suh J. M., Hah N. (2013). PPARγ signaling and metabolism: the good, the bad and the future. *Nature Medicine*.

[83] Xu Y. Y., Takahashi Y., Wang Y. (2009). Downregulation of *GATA-2* and overexpression of adipogenic gene-PPARγ in mesenchymal stem cells from patients with aplastic anemia. *Experimental Hematology*.

[84] Wang H. Y., Ding T. L., Xie Y., Xu X. P., Yu L., Chen T. (2009). Osteogenic and adipogenic differentiation of bone marrow-derived mesenchymal stem cells in patients with aplastic anemia. *Zhonghua Nei Ke Za Zhi*.

[85] Jiang S. Y., Xie X. T., Zhou J. J. (2009). Gene profile and fibroblast growth factor 2 expression in mesenchymal stem cell from children with aplastic anemia. *Zhonghua Er Ke Za Zhi*.

[86] Jiang S. Y., Xie X. T., Jiang H., Zhou J. J., Li F. X., Cao P. (2014). Low expression of basic fibroblastic growth factor in mesenchymal stem cells and bone marrow of children with aplastic anemia. *Pediatric Hematology and Oncology*.

[87] Madrigal M., Rao K. S., Riordan N. H. (2014). A review of therapeutic effects of mesenchymal stem cell secretions and induction of secretory modification by different culture methods. *Journal of Translational Medicine*.

[88] Yeoh J. S. G., van Os R., Weersing E. (2006). Fibroblast growth factor-1 and -2 preserve long-term repopulating ability of hematopoietic stem cells in serum-free cultures. *Stem Cells*.

[89] Li J., Lu S., Yang S. (2012). Impaired immunomodulatory ability of bone marrow mesenchymal stem cells on CD4^+^ T cells in aplastic anemia. *Results in Immunology*.

[90] Bueno C., Roldan M., Anguita E. (2014). Bone marrow mesenchymal stem cells from patients with aplastic anemia maintain functional and immune properties and do not contribute to the pathogenesis of the disease. *Haematologica*.

[91] Morley A., Blake J. (1974). Animal-model of chronic aplastic marrow failure .I. Late marrow failure after busulfan. *Blood*.

[92] Pugsley C. A. J., Forbes I. J., Morley A. A. (1978). Immunological abnormalities in an animal-model of chronic hypoplastic marrow failure induced by busulfan. *Blood*.

[93] Andrews C. M., Spurling N. W., Turton J. A. (1993). Characterization of busulfan-induced myelotoxicity in b6c_3_f_1_ mice using flow-cytometry. *Comparative Haematology International*.

[94] Andrews C. M., Williams T. C., Turton J. A. (1998). Long-term haematological alterations in female B6C_3_F_1_ mice treated with busulphan. *Comparative Haematology International*.

[95] Gibson F. M., Andrews C. M., Diamanti P. (2003). A new model of busulphan-induced chronic bone marrow aplasia in the female BALB/c mouse. *International Journal of Experimental Pathology*.

[96] Liu L. P., Liu J. F., Lu Y. Q. (2001). Effects of Sheng-Mai injection on the PRPP synthetase activity in BFU-Es and CFU-Es from bone marrows of mice with benzene-induced aplastic anemia. *Life Sciences*.

[97] Lezama R. V., Escorcia E. B., Torres A. M. (2001). A model for the induction of aplastic anemia by subcutaneous administration of benzene in mice. *Toxicology*.

[98] Huang Y. L., Huang S. L., Cai Y. (2007). Effect of bone marrow mesenchymal stem cell infusion on hematopoiesis in mice with aplastic anemia. *Zhongguo Shi Yan Xue Ye Xue Za Zhi*.

[99] Zhou Y.-M., Huang T., Xue Z.-Z., Huang Z.-Q. (1998). Experimental study on mice with immune-mediated aplastic anemia. *Journal of Experimental Hematology*.

[100] Li J., Chen H., Lv Y. B. (2016). Intraperitoneal injection of multiplacentas pooled cells treatment on a mouse model with aplastic anemia. *Stem Cells International*.

[101] Barnes D. W., Mole R. H. (1967). Aplastic anaemia in sublethally irradiated mice given allogeneic lymph node cells. *British Journal of Haematology*.

[102] Huang L. F., Zhou K., Zhang N. (2009). Establishment of a bone marrow failure mouse model by immune injury. *Zhonghua Xue Ye Xue Za Zhi*.

[103] Renz J. F., Kalf G. F. (1991). Role for interleukin-1 (IL-1) in benzene-induced hematotoxicity: Inhibition of conversion of pre-IL-1 alpha to mature cytokine in murine macrophages by hydroquinone and prevention of benzene-induced hematotoxicity in mice by IL-1 alpha. *Blood*.

[104] Peng R., Wang D., Wang B. (1999). Apoptosis of hemopoietic cells in irradiated mouse bone marrow. *Journal of Environmental Pathology, Toxicology and Oncology*.

[105] Dainiak N. (2002). Hematologic consequences of exposure to ionizing radiation. *Experimental Hematology*.

[106] Hall E. J., Giaccia A. J. (2012). *Radiobiology for the Radiologist*.

[107] Cui Y. F., Gao Y. B., Yang H., Xiong C. Q., Xia G. W., Wang D. W. (1999). Apoptosis of circulating lymphocytes induced by whole body gamma-irradiation and its mechanism. *Journal of Environmental Pathology, Toxicology and Oncology*.

[108] Singh V. K., Newman V. L., Berg A. N., MacVittie T. J. (2015). Animal models for acute radiation syndrome drug discovery. *Expert Opinion on Drug Discovery*.

[109] Young N. S., Bacigalupo A., Marsh J. C. (2010). Aplastic anemia: pathophysiology and treatment. *Biology of Blood and Marrow Transplantation*.

[110] Marsh J. C. W. (2005). Management of acquired aplastic anaemia. *Blood Reviews*.

[111] Klingemann H., Matzilevich D., Marchand J. (2008). Mesenchymal stem cells - sources and clinical applications. *Transfusion Medicine and Hemotherapy*.

[112] Armand P., Antin J. H. (2007). Allogeneic stem cell transplantation for aplastic anemia. *Biology of Blood and Marrow Transplantation*.

[113] Gordon-Smith T. (2009). Aplastic anaemia and bone marrow failure. *Medicine*.

[114] Qiao S. K., Ren H. Y., Shi Y. J., Liu W. (2014). Allogeneic compact bone-derived mesenchymal stem cell transplantation increases survival of mice exposed to lethal total body irradiation: a potential immunological mechanism. *Chinese Medical Journal*.

[115] Fernandez-Garcia M., Yanez R. M., Sanchez-Dominguez R. (2015). Mesenchymal stromal cells enhance the engraftment of hematopoietic stem cells in an autologous mouse transplantation model. *Stem Cell Research & Therapy*.

[116] Zhang J. M., Zhou S. Y., Zhou Y. (2016). Adipose-derived mesenchymal stem cells (ADSCs) with the potential to ameliorate platelet recovery, enhance megakaryopoiesis, and inhibit apoptosis of bone marrow cells in a mouse model of radiation-induced thrombocytopenia. *Cell Transplantation*.

[117] Rasmusson I. (2006). Immune modulation by mesenchymal stem cells. *Experimental Cell Research*.

[118] LeBlanc K., Frassoni F., Ball L. (2008). Mesenchymal stem cells for treatment of steroid-resistant, severe, acute graft-versus-host disease: a phase II study. *Lancet*.

[119] Ringden O. (2009). Mesenchymal stromal cells as first-line treatment of graft failure after hematopoietic stem cell transplantation. *Stem Cells and Development*.

[120] Fouillard L., Bensidhoum M., Bories D. (2003). Engraftment of allogeneic mesenchymal stem cells in the bone marrow of a patient with severe idiopathic aplastic anemia improves stroma. *Leukemia*.

[121] Jaganathan B. G., Tisato V., Vulliamy T. (2010). Effects of MSC co-injection on the reconstitution of aplastic anemia patient following hematopoietic stem cell transplantation. *Leukemia*.

[122] Chao Y. H., Tsai C., Peng C. T. (2011). Cotransplantation of umbilical cord MSCs to enhance engraftment of hematopoietic stem cells in patients with severe aplastic anemia. *Bone Marrow Transplantation*.

[123] Xiao Y., Jiang Z. J., Pang Y. (2013). Efficacy and safety of mesenchymal stromal cell treatment from related donors for patients with refractory aplastic anemia. *Cytotherapy*.

[124] Si Y. J., Yang K., Qin M. Q. (2014). Efficacy and safety of human umbilical cord derived mesenchymal stem cell therapy in children with severe aplastic anemia following allogeneic hematopoietic stem cell transplantation: a retrospective case series of 37 patients. *Pediatric Hematology and Oncology*.

[125] Cle D. V., Santana-Lemos B., Tellechea M. F. (2015). Intravenous infusion of allogeneic mesenchymal stromal cells in refractory or relapsed aplastic anemia. *Cytotherapy*.

[126] Guangzhou General Hospital of Guangzhou Military Command, Guangzhou Municipal Twelfth People’s Hospital and Guangdong Prevention and Treatment Center for Occupational Diseases (2017). Mesenchymal stem cells transplantation to patients with relapsed/refractory aplastic anemia. http://clinicaltrials.gov/ct2/show/record/NCT01305694.

[127] University of Sao Paulo, Cle, D.V (2017). Bone marrow mesenchymal stem cells in the treatment of refractory severe acquired aplastic anemia. https://clinicaltrials.gov/ct2/show/record/NCT01297972.

[128] Meuleman N., Tondreau T., Ahmad I. (2009). Infusion of mesenchymal stromal cells can aid hematopoietic recovery following allogeneic hematopoietic stem cell myeloablative transplant: a pilot study. *Stem Cells and Development*.

[129] Waselenko J. K., MacVittie T. J., Blakely W. F. (2004). Medical management of the acute radiation syndrome: recommendations of the strategic national stockpile radiation working group. *Annals of Internal Medicine*.

[130] Führer M., Rampf U., Baumann I. (2005). Immunosuppressive therapy for aplastic anemia in children: a more severe disease predicts better survival. *Blood*.

[131] Rosenfeld S., Follmann D., Nunez O., Young N. S. (2003). Antithymocyte globulin and cyclosporine for severe aplastic anemia: association between hematologic response and long-term outcome. *JAMA*.

[132] Buron F., Perrin H., Malcus C. (2009). Human mesenchymal stem cells and immunosuppressive drug interactions in allogeneic responses: an in vitro study using human cells. *Transplantation Proceedings*.

